# Internet-delivered cognitive behavioral interventions to reduce elevated stress: A systematic review and meta-analysis

**DOI:** 10.1016/j.invent.2022.100553

**Published:** 2022-06-22

**Authors:** Frank Svärdman, Douglas Sjöwall, Elin Lindsäter

**Affiliations:** a Center for Psychiatry Research, Department of Clinical Neuroscience, Karolinska Institutet, Stockholm, Sweden; b Center for Psychiatry Research, Region Stockholm, Center for Neurodevelopmental Disorders at Karolinska Institutet (KIND), CAP Research Center, Gävlegatan 22B, SE-11330 Stockholm, Sweden; c Department of Women's and Children's Health, Center for Neurodevelopmental Disorders at Karolinska Institutet (KIND), Karolinska Institutet, Stockholm, Sweden; d Habilitation & Health, Stockholm Health Care Services, Region Stockholm, Sweden; e Division of Psychology, Department of Clinical Neuroscience, Karolinska Institutet, Stockholm, Sweden

**Keywords:** Psychological stress, Internet, Intervention, Cognitive behavioral therapy

## Abstract

**Background:**

Face-to-face cognitive behavioral therapy (CBT) is the most promising treatment to reduce stress, but access to CBT is limited. Internet-delivered CBT (ICBT) enables large-scale dissemination at low costs. Evidence suggests that ICBT can reduce stress in subclinical and mixed diagnostic samples, but less is known about the effect of ICBT in targeted samples suffering from elevated perceived stress or stress-related disorders.

**Objective:**

To investigate the efficacy of ICBT specifically aimed at reducing stress in adults with elevated perceived stress or stress-related disorders.

**Methods:**

We searched for randomized controlled trials comparing ICBT with a control group in PubMed, Web of Science, and PsycInfo between 2010 and 2021. A meta-analysis of 14 comparisons (total *N* = 1831) was performed, and Cohen's *d* was calculated to assess the difference between intervention and control groups at posttest for the primary outcome self-rated stress. Effects on secondary outcomes of anxiety and depression were also investigated.

**Results:**

The pooled mean effect size for self-rated stress at posttest was *d* = 0.78 [CI 95 % 0.66–0.90]. For anxiety and depression, the effects were *d* = 0.69 [95 % CI 0.52–0.86] and *d* = 0.65 [95 % CI 0.56–0.75] respectively. The heterogeneity of results between studies was overall low to moderate. Subgroup analyses were not conducted due to the limited number of studies eligible for inclusion.

**Conclusions:**

Results provide evidence of the efficacy of ICBT to reduce stress, anxiety, and depressive symptoms in adults suffering from elevated stress or stress-related disorders. Findings have important implications for the development of safe and evidence-based treatment guidelines in the face of a rapid digital expansion.

This study was preregistered at Open Science Framework (osf.io) with DOI 10.17605/OSF.IO/BQAZ3.

## Highlights

• First review and meta-analysis to investigate ICBT in targeted samples with elevated stress or stress-related disorders.
• Thirteen included studies indicate that ICBT can reduce perceived stress as well as anxiety- and depression symptoms.
• More studies are needed to strengthen the evidence for ICBT for stress-reduction.

## 1 Introduction

Stress, defined as psychophysiological activation in response to perceived threat or challenge, is necessary for our survival and for adaptation to an ever-changing environment (McEwen, 1998). Elevated stress over prolonged periods, however, can have a negative impact on a range of biological systems (O'Connor et al., 2021) and is associated with adverse physical and psychological sequelae such as depressed mood, anxiety, impaired sleep, exhaustion, and functional impairment (Cohen et al., 2007; Grossi et al., 2015; McEwen, 2006). Total costs of stress in the western world have been estimated up to USD 187 billion, primarily attributable to a high incidence of sickness absence, productivity loss, and increased healthcare consumption (Hassard et al., 2018).

Even though prolonged elevated stress can have serious negative implications for afflicted individuals and for society as a whole, there is a lack of international consensus regarding how to operationalize and diagnose symptoms of stress (Grossi et al., 2015; Lindsäter et al., 2022). Further, there is ongoing debate regarding how these symptoms can be demarcated from other psychiatric conditions and to adaptive reactions to stressful life events (Bianchi et al., 2021; Casey, 2021; Koutsimani et al., 2019). The International Classification of Diseases (ICD) (World Health Organization, 1992) and the Diagnostic and Statistical Manual of Mental Disorders (DSM) (American Psychiatric Association, 2013) devote sections to stress-related disorders and state that these can be set for individuals with clinically significant suffering and/or functional impairment that has a clear association to identifiable acute or chronic stressful life events (e.g., relational conflicts, economic hardship, loss of a loved one, or work-related stressors). The symptomatic development should not be better explained by other psychiatric or somatic conditions. One of these diagnoses, adjustment disorder, is amongst the most commonly used psychiatric diagnoses globally (Evans et al., 2013), with prevalence estimates of 11.5 % in outpatient psychiatric clinics (Yaseen, 2017) and 9.2 % in primary care settings (Sundquist et al., 2017). The diagnosis exhaustion disorder, similar to the construct of clinical burnout (van Dam, 2021), is characterized by severe mental and physical fatigue in the wake of persistent exposure to sub-traumatic stressors, and is the most common cause for sick leave of all psychiatric and somatic disorders in Sweden (The Swedish Social Insurance Agency, 2020). In clinical research, however, diagnostic labels such as adjustment disorder, exhaustion disorder, neurasthenia, and burnout are often used interchangeably, and are commonly assumed to describe similar clinical pictures (Grossi et al., 2015; Arends et al., 2012). In the present study, these conditions will collectively be referred to as “stress-related disorders”.

Meta-analyses of face-to-face interventions to prevent or reduce stress in the working population jointly indicate that interventions based on cognitive behavioral therapy (CBT) can be effective in reducing perceived stress with moderate to large effect sizes compared to controls (van der Klink et al., 2001; Richardson and Rothstein, 2008; Bhui et al., 2012; Estevez Cores et al., 2021). Importantly, prior studies have mainly investigated preventive stress-management interventions using universally recruited, subclinical samples. Randomized controlled trials of CBT for targeted samples (i.e., when individuals are selected based on certain inclusion criteria) reporting elevated perceived stress or suffering from stress-related disorders are few and results are inconclusive (Arends et al., 2012; Ahola et al., 2017; Perski et al., 2017; Maricutoiu et al., 2016). Given that individuals with higher symptom burden and functional disability are the ones that are most likely to seek healthcare, the lack of evidence for treatment is troublesome. Finding highly accessible and efficient treatments to reduce the clinical impact of stress is important.

The past decades have seen a rapid development of digital solutions for providing psychological treatments, and there is no indication that this development will subside (Robbins et al., 2020). It is important that the development is grounded on scientific evidence to secure high-quality care. Indeed, CBT when delivered via the internet (ICBT) has been found to be effective in the treatment of anxiety disorders, depression, and insomnia while simultaneously reducing therapist time and enabling increased accessibility of care for patients (Andersson et al., 2019). When it comes to treatments targeting perceived stress and stress-related disorders the evidence-base is less established. An abundance of theoretically sprawling internet-delivered stress-management interventions have been developed, most often preventative in nature using universally recruited samples (Heber et al., 2017; Stratton et al., 2017; Carolan et al., 2017; Ryan et al., 2017). In a meta-analysis by Heber et al. (2017), subgroup analyses of ICBT aimed at reducing stress yielded small to moderate effect sizes compared to controls. Given that face-to-face CBT is indicated to be effective in reducing perceived stress, and the evidence of ICBT to reduce symptoms that commonly overlap with stress-related disorders (e.g., anxiety, depressed mood, and disturbed sleep), further investigation into the effect of ICBT for targeted samples reporting elevated stress and suffering from stress-related disorders is warranted to ensure the development of high-quality care for this large group of individuals.

The aim of this study was to conduct an up-to-date systematic review and meta-analysis of internet-delivered interventions based on CBT to evaluate treatment efficacy in targeted (as opposed to universal) adult populations reporting elevated perceived stress or diagnosed with stress-related disorders.

## 2 Methods

This systematic review and meta-analysis was conducted in accordance with PRISMA (Preferred Reported Items for Systematic Review and Meta-analysis) guidelines (Page et al., 2021). This study was preregistered in the Open Science Framework (OSF) and can be retrieved at 10.17605/OSF.IO/BQAZ3.

### 2.1 Eligibility criteria

Randomized controlled trials that were published in English between 2010 and 2021 in a peer-reviewed journal were eligible for inclusion, given that the following criteria were met according to pre-established PICO (population, intervention, control, outcome): (1) Adult population (≥18 years) with elevated perceived stress or stress-related disorder; (2) the study investigates the effect of internet-delivered cognitive and/or behavioral interventions specifically aimed at reducing stress (3) compared to a waitlist control, active control, or treatment as usual; (4) a primary or secondary outcome measuring self-rated stress.

Because there are no established criteria for how to assess elevated stress, we included studies that used targeted recruitment (participants were either self-referred or clinically referred to the study) with inclusion criteria based on a cut-off on an established stress-scale (e.g., the Perceived Stress Scale (Cohen et al., 1983)) or structured clinical assessment to establish a stress-related disorder. Stress-related disorders were operationalized as sub-traumatic stress-disorders as defined in the ICD or in the DSM, as well as burnout and neurasthenia. “Internet-delivered” was defined as any intervention that was administered only via a computer or internet-connected device. In other words, blended treatment approaches (i.e., combining face-to-face therapy with the internet-based intervention) were not included. Cognitive behavioral treatments were defined as interventions based on cognitive principles (e.g., cognitive restructuring), behavior principles (e.g., behavioral activation, exposure), or a combination of both. Studies that primarily addressed the general well-being of participants, in which stress was one of many outcomes, were not included. In cases where mixed samples of participants were studied (e.g., depression, anxiety, and stress), at least 60 % of study participants needed to explicitly suffer from a stress-related disorder. Interventions targeting participants with other primary somatic or psychiatric conditions (e.g., cancer, headache, depression, posttraumatic stress disorder) or caregivers of people with medical conditions (e.g., caregivers for dementia) were excluded.

### 2.2 Information sources and search strategy

Searches were conducted in PubMed, Web of Science, and PsycInfo by combining four categories of search terms; (1) stress, (2) CBT as the intervention, (3) RCT as the design, and (4) internet as the context of the study. The search strategy was developed with assistance from a research librarian at the Karolinska University Library. Due to the rapid technological advancements and accessibility to internet in recent years, the search was restricted to studies published since the year 2010. No restrictions were imposed on searches regarding publication status or language. The exact search can be found in Table S1 in the Online supplementary material. The first search was conducted between June and July 2020 and was repeated in all databases in May 2021 to ensure that the most recent relevant studies were included. Reference lists of all included studies and previous relevant meta-analyses and reviews were hand-searched. All studies from the searches were imported into Rayyan (https://rayyan.qcri.org/), a web-based tool for screening and selecting studies in systematic reviews.

### 2.3 Study selection

After removing duplicates, the eligibility of the studies was assessed in two stages. First, two of the authors (DS and FS) independently included or excluded search hits based on titles and abstracts. Any disagreement or uncertainty regarding study eligibility in this stage was discussed with the third author (EL), and a decision was made in consensus. In the second stage, the three authors assessed one third each of the full-text articles for eligibility, with frequent meetings to discuss any ambiguities.

### 2.4 Data extraction

For the final set of included studies, data regarding study characteristics (first author, country, sample size, participant age and sex, type of control group, outcomes, time of follow-up, and drop-out rates) were extracted to an Excel file, as were data regarding intervention characteristics (treatment content, type of guidance, and length of intervention). Pre- and post-intervention means and standard deviations for the primary outcome stress as well as, when available, depression and anxiety outcomes, were extracted for meta-analysis. In cases of missing information or ambiguities in reported outcomes, principal investigators of respective studies were contacted to provide complementary information. This occurred in three cases and all investigators replied.

### 2.5 Risk of bias assessment

The risk of bias of each included study was assessed using five dimensions of the revised Cochrane risk of bias tool for randomized trials (Rob2) (Sterne et al., 2019a): (1) Risk arising from the randomization process, (2) deviations from the intended interventions, (3) missing outcome data, (4) measurement of the outcome, and (5) selection of the reported result. Bias related to blinding was not assessed due to difficulties in clinical trials of psychological interventions to blind both therapists and participants. Studies were graded as “low risk”, “some concerns”, or “high risk”, resulting in an overall bias assessment. Two authors, DS and FS, independently rated all articles. Conflicting ratings were discussed and resolved in consensus.

### 2.6 Statistical analyses

All analyses were conducted using the statistical software Cochrane Rev Man 5 (v.5.3). The primary statistical analysis was a meta-analysis of between-group effects with self-rated stress as the outcome, using the post-intervention mean and standard deviation. When available, measures of anxiety and depression were analyzed using the same method as for the primary outcome. If a study investigated more than one modality of treatment intervention versus a control group, the different treatments were included as separate comparisons versus the control. Follow-up data from post-intervention assessment to follow-up assessments was also analyzed as a within-group effect when available. Because heterogeneity between studies was expected, a random-effects model was used. Heterogeneity was quantified based on the *I*^2^ statistic. This represents the proportion of the variance between studies that can be attributed to real study differences in effect and is measured in percentages. Confidence intervals for the *I*^2^ statistic were calculated based on recommendations of Higgins and Thompson (2002). An *I*^2^ value of 25 % is considered low heterogeneity, 50 % as moderate, and 75 % as high (Higgins et al., 2003). Pooled effect sizes were calculated using Cohen's *d*, where *d* = 0.2 is considered a small effect, *d* = 0.5 as a medium effect, and *d* = 0.8 as a large effect.

Publication bias was assessed by constructing a funnel plot for the primary outcome of self-rated stress. As a more objective complement to visual inspection, Egger's regression test was used to measure plot asymmetry (Egger et al., 1997). In addition, publication bias was also tested assuming homogeneity and heterogeneity using the software JASP (v. 014.1.0).

To complement the main analysis with more in-depth information about treatment factors that have previously been found to affect outcomes in internet-delivered interventions for stress-reduction (Heber et al., 2017), a priori aim was to conduct subgroup analyses of type of guidance, treatment length, and study quality (i.e., risk of bias). According to recommendations from Fu et al. (2011), subgroup analyses should only be performed if each subgroup contains a minimum of six studies to avoid uneven distributions. Hence, no subgroup analyses were conducted in the present study. In addition to the preregistered analyses, we conducted exploratory post-hoc analyses of secondary outcomes related to exhaustion and insomnia using the same methods as described for the primary outcome. Mental and physical exhaustion and disturbed sleep are common symptoms of stress-related disorders (Grossi et al., 2015), and reduced insomnia severity has previously been found to mediate the effect of ICBT on perceived stress and symptoms of exhaustion (Lindsäter et al., 2021).

## 3 Results

### 3.1 Study selection

**Fig. 1:**
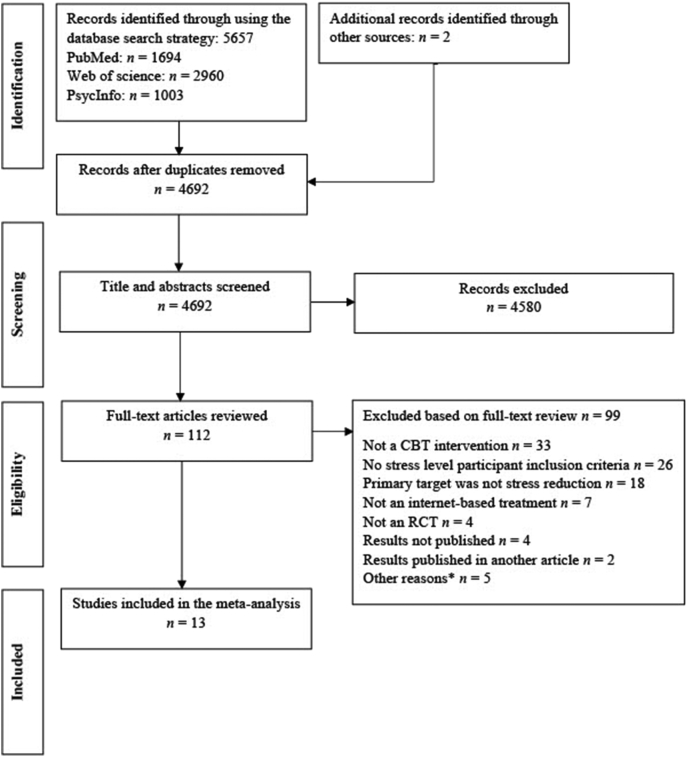
PRISMA flowchart illustrating the study selection process. CBT, cognitive behavior therapy; RCT, randomized controlled trial. *Other reasons: e.g. full article not being published online or not peer-reviewed.

Fig. 1 illustrates a flowchart of the study selection process. Thirteen studies were included in the final sample, consisting of 14 separate comparisons. Of note, one study in which authors labeled the intervention “blended CBT” (Leterme et al., 2020) was included because the face-to-face component to the internet-delivered intervention (5 min before and 5 minafter each module, consisting of adherence-focused guidance and technical support) was not deemed to qualify as therapeutic support.

### 3.2 Study characteristics

Table 1 presents an overview of the trial characteristics of the 13 included studies. More than half of the trials were conducted in Germany (Ebert et al., 2016a; Ebert et al., 2016b; Heber et al., 2016; Harrer and Adam, 2018; Jonas et al., 2016; Nixon et al., 2021; Mehring et al., 2016), two in Sweden (Persson Asplund et al., 2018; Lindsäter et al., 2018), and one in Lithuania (Eimontas et al., 2018), France (Leterme et al., 2020), the United States (Rose et al., 2013), and Spain (Rachyla et al., 2018), respectively. Ten of 13 studies were conducted in a research setting, whereas three were conducted in healthcare settings such as online coaching service (Jonas et al., 2016), psychiatric consultation service (Leterme et al., 2020), and primary healthcare clinic (Mehring et al., 2016). In total, post-treatment data from 1831 participants (ICBT, *n* = 909; control groups, *n* = 922) were available for the analysis of the main outcome of self-rated stress. The most common type of control condition was waitlist control (*n* = 10). Two studies used an attention control group, both consisting of an educational intervention giving participants information about stress, and one study compared ICBT to treatment as usual. In one study, participants were randomized to either adherence-guided ICBT, self-guided ICBT, or a waitlist control condition (Nixon et al., 2021).

**Table 1:** Interventions characteristics of included studies (*N* = 13).

Study	Origin^a^	Study setting	N	Controls^b^	Outcomes					Follow-up from pre-assessment weeks (w); months (m)
					Stress^c^	Anxiety^d^	Depression^e^	Exhaustion^f^	Insomnia^g^	
Ebert et al. (2016)^1^	Ger	Research	264	WL	PSS-10	HADS-A	CES-D	MBI-EE	ISI	7 w; 6 m
Ebert et al. (2016)^2^	Ger	Research	264	WL	PSS-10	HADS-A	CES-D	MBI-EE	ISI	7 w; 6 m
Eimontas et al. (2018)^3^	Lit	Research	284	WL	ADNM-8	–	–	–	–	4 w
Harrer et al. (2018)^4^	Ger	Research	150	WL	PSS-4	SSTA-I	CES-D	MBI-EE	–	7 w; 3 m
Heber et al. (2016)^5^	Ger	Research	264	WL	PSS-10	HADS-A	CES-D	MBI-EE	ISI	7 w; 6 m; 12 m
Jonas et al. (2016)^6^	Ger	Clinical	39	WL	DASS-21	DASS-21	DASS-21	MBI-EE	–	12 w; 6 m; 12 m
Leterme et al. (2020)^7^	Fra	Clinical	120	WL	PSS-14	HADS-A	HADS	–	–	8 w; 6 m
Lindsäter et al. (2018)^8^	Swe	Research	100	WL	PSS-14	GAD-7	MADRS-S	SMBQ	ISI	12 w; 9 m
Mehring et al. (2016)^9^	Ger	Clinical	93	TAU	PSQ	–	–	–	–	12 w
Nixon et al. (2021)^10^	Ger	Research	404	WL/SG	PSS-10	–	CES-D	MBI-EE	–	7 w; 6 m
Persson-Asplund et al. (2018)^11^	Swe	Research	117	AC	PSS-14	–	MADRS-S	SMBQ	ISI	8 w; 6 m
Rachyla et al. (2020)^12^	Spa	Research	68	WL	ISL	BAI	BDI-II	–	–	12 w; 6 m; 12 m
Rose et al. (2013)^13^	US	Research	66	AC	PSS-10	–	–	–	–	6 w

a Swe, Sweden; Ger, Germany; Lit, Lithuania; Fra, France; US, United States; Spa, Spain.

b WL, Waiting list; TAU, treatment as usual; SG, Self-guided; AC, Attention control

c PSS, Perceived Stress Scale; ADNM, Adjustment disorder new module; DASS; Depression Anxiety Stress Scales; PSQ, Perceived Stress Questionnaire; ISL, Inventory of Stress and Loss; BAI, Beck Anxiety Inventory.

d DASS; Depression Anxiety Stress Scales; HADS, Hospital Anxiety and Depression Scale; SSTA-I, Spielberger State-Trait Anxiety Inventory; GAD-7, Generalized Anxiety Disorder 7-item scale; BAI, Beck Anxiety Inventory.

e CES-D, Center for Epidemiological Studies Depression Scale; DASS; Depression Anxiety Stress Scales; HADS, Hospital Anxiety and Depression Scale; MADRS-S, Montgomery Åsberg Depression Rating Scale; BDI-II, Beck Depression Inventory.

f MBI-EE; Emotional Exhaustion Subscale of the Maslach Burnout Inventory, SMBQ; Shirom-Melamed Burnout Questionnaire.

g ISI; Insomnia Severity Index.

The most common measurement of stress was the Perceived Stress Scale (PSS-10 or PSS-14), which was used as a primary outcome measure in eight studies and as a secondary outcome in one study. Beyond measurement of stress, symptoms of anxiety were assessed in eight studies and depression was assessed in ten studies. Eight studies (nine comparisons) assessed exhaustion and five studies assessed insomnia.

Three studies provided no follow-up data after post-intervention assessment (Mehring et al., 2016; Eimontas et al., 2018; Rose et al., 2013). The remaining studies provided follow-up data after 3 months (Harrer and Adam, 2018; Jonas et al., 2016; Rachyla et al., 2018) 6 months (Leterme et al., 2020; Ebert et al., 2016a; Ebert et al., 2016b; Heber et al., 2016; Jonas et al., 2016; Nixon et al., 2021; Persson Asplund et al., 2018; Rachyla et al., 2018), 9 months (Lindsäter et al., 2018), and 12 months (Heber et al., 2016; Jonas et al., 2016; Rachyla et al., 2018) (from pre-assessment dates).

### 3.3 Recruitment procedures and participant characteristics

Table S2 in the Online supplement presents an overview of participant characteristics including main inclusion- and exclusion criteria for each included study. Nine of 13 studies recruited participants through advertisement to the general population, to occupational healthcare, and to insurance companies. Two studies particularly targeted university students (Harrer and Adam, 2018; Rose et al., 2013), and two studies recruited participants via clinical referral (Leterme et al., 2020; Mehring et al., 2016). In recruiting participants, eight studies used a stress scale cut-off, of which six used versions of the Perceived Stress Scale (Ebert et al., 2016a; Ebert et al., 2016b; Heber et al., 2016; Harrer and Adam, 2018; Nixon et al., 2021; Rose et al., 2013), one used the Maslach Burnout Inventory (MBI) (Jonas et al., 2016), and one used the Brief Adjustment Disorder New Model (ADNM-8) questionnaire (Eimontas et al., 2018). In five studies, clinical assessment was used for inclusion (Leterme et al., 2020; Mehring et al., 2016; Persson Asplund et al., 2018; Lindsäter et al., 2018; Rachyla et al., 2018) of which all but one (Mehring et al., 2016) assessed criteria for adjustment disorder or exhaustion disorder (both classified under F43 *Reactions to severe stress, and adjustment disorders* in the ICD).

A majority of study participants were women (84 %; 1547/1842), and mean ages of study samples ranged from 24 (Harrer and Adam, 2018) to 47 (Jonas et al., 2016) years with a median mean age of 42 years across studies. Education level was high with 63–100 % of participants across studies reporting college or university studies. Only seven studies reported sick leave in their samples, of which six reported sick leave rates of 1–3 % and one study reported that 14 % were on sick leave upon commencing treatment (Lindsäter et al., 2018).

### 3.4 Intervention characteristics

Table S3 in the Online supplement presents an overview of intervention characteristics. The mean length of treatment across studies was 7.4 weeks (median = 7 weeks; range = 4 to 12 weeks). In all but one study (Leterme et al., 2020), interventions were available on a computer through the internet and five interventions were developed to work equally well on a smart mobile phone (Ebert et al., 2016a; Ebert et al., 2016b; Heber et al., 2016; Harrer and Adam, 2018; Nixon et al., 2021). In the study by Leterme et al (Leterme et al., 2020), the intervention was administered via a computer on-site, with each session delivered via a USB key.

#### 3.4.1 Treatment content

All treatments were presented to participants as a number of sessions referred to as modules. Most treatments required participants to have finished one module before the next was made available, but in the study by Eimontas et al (Eimontas et al., 2018) all modules were available from the start. Four studies investigated the effect of the same intervention (GET.ON Stress) (Ebert et al., 2016a; Ebert et al., 2016b; Heber et al., 2016; Nixon et al., 2021), and one additional study was based on GET.ON Stress but included some additional components of third-wave CBT such as self-compassion and acceptance (Harrer and Adam, 2018). In six studies (Ebert et al., 2016a; Ebert et al., 2016b; Heber et al., 2016; Harrer and Adam, 2018; Nixon et al., 2021; Persson Asplund et al., 2018), optional modules could be chosen by participants in addition to pre-selected content. Though the order and content of treatment modules varied somewhat across treatments, several interventions recurred. The most common treatment modules were: “Psychoeducation” (i.e., information about the nature of stress and common reactions to stress), relaxation techniques (e.g., breathing exercises, applied relaxation), problem-solving techniques, coping strategies (e.g., emotion regulation), behavioral activation, exposure techniques (targeting, e.g., worry and rumination, perfectionism), and cognitive reappraisal techniques (including psychological detachment and cognitive flexibility). Interventions targeting improved sleep were available in seven treatment programs, some type of social focus (i.e., communication skills, strengthening relationships) was available in six programs, and mindfulness exercises were included in four.

#### 3.4.2 Treatment guidance

Five types of guidance were identified in the included studies. Four interventions used a combination of written reminders and feedback by a therapist on request, referred to as adherence-focused guidance (AG) (Heber et al., 2017; Ebert et al., 2016b; Harrer and Adam, 2018; Nixon et al., 2021). In the study by Leterme et al. (2020) adherence-focused guidance was administered by a short chat with a nurse. Four interventions (Jonas et al., 2016; Persson Asplund et al., 2018; Lindsäter et al., 2018; Rachyla et al., 2018) used weekly written guidance by a therapist, referred to as weekly guidance (WL). Two interventions used computer-automated guidance (i.e., without therapist involvement) (Ebert et al., 2016a; Mehring et al., 2016), and one study tested a self-guided intervention with reminders via mail and telephone (Rose et al., 2013). Two interventions were completely self-guided (Nixon et al., 2021; Eimontas et al., 2018).

### 3.5 Risk of bias within studies

**Fig. 2:**
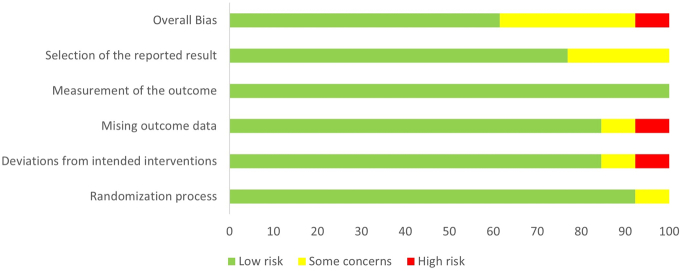
Overview of risk of bias assessment according to the revised Cochrane risk of bias tool for randomized trials (Rob2) (Sterne et al., 2019). Each colored area represents the percentage of studies in the respective bias assessments categories.

Fig. 2 shows an overview of the risk of bias assessment. A more detailed description of the risk of bias assessment for each study can be found in the Online supplement (Fig. S1). Eight studies were assessed to have an overall low risk of bias. Four studies were assessed to have “some concerns” regarding their overall bias. The most common reason for rating “some concern” was in the selection of the reported result, due to lack of pre-registration of the study. Only one study was rated as having an overall high risk of bias. Attrition rate varied widely across studies (range 3 % to 69 %). Eleven studies based their analyses on the intention-to-treat principle, whereas two studies only presented per-protocol analysis.

### 3.6 Post-treatment effects on the level of stress, anxiety, and depression

**Fig. 3:**
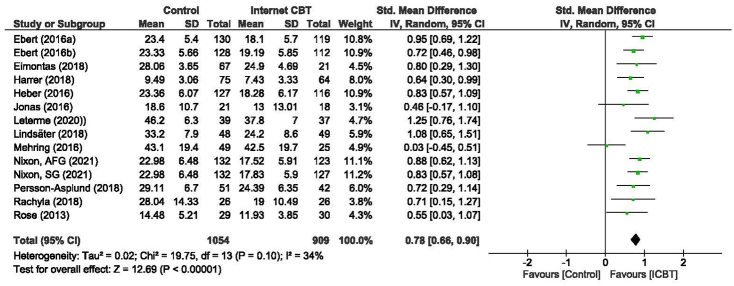
Forest plot of post-intervention effect sizes of all included Internet-delivered cognitive behavioral interventions (ICBT) compared to control conditions, on the main outcome of self-rated stress. AFG, adherence focused guidance. SG, self-guided.

**Fig. 4:**
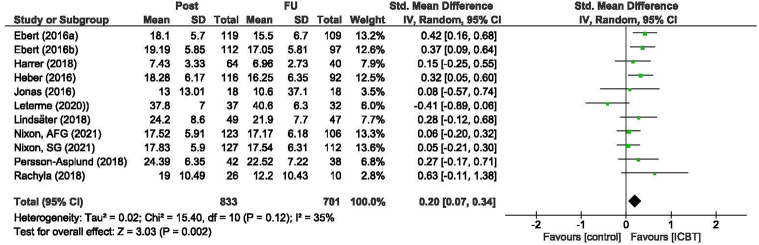
Forest plot of within-group effect size based on post-intervention means and standard deviations compared with last follow-up means and standard deviations on the main outcome of self-rated stress. AFG, adherence focused guidance. SG, self-guided.

As shown in Fig. 3, the pooled effect size for the primary outcome stress was *d* = 0.78 [95 % CI 0.66–0.9] in favor of ICBT compared to control conditions. Heterogeneity was low to moderate (*I*^2^ = 34 % [95 % CI 25 %–65 %]). Within-group follow-up effects from post-intervention assessment to follow-up assessments were reported in 11 comparisons from 10 studies (see Fig. 4). In cases where multiple follow-ups were conducted, the last assessment point was chosen. The within-group effect size, Cohen's *d* = 0.2 [95 % CI 0.07–0.33] indicated stability or a slight increase in the effect of the ICBT over time. Heterogeneity was low to moderate (*I*^2^ = 35 % [95 % CI 32 %–68 %]).

Figs. S2 and S3 in the Online supplement show forest plots of post-intervention effect sizes of included studies on the secondary outcomes of self-rated anxiety and depression respectively. Symptoms of anxiety were assessed in eight studies and yielded a pooled post-treatment effect size of Cohen's *d* = 0.69 [95 % CI 0.52–0.86], with a low to moderate heterogeneity (*I*^2^ = 44 % [95 % CI 28 %–75 %]). Ten studies with a total of 11 comparisons assessed depression symptoms, generating a pooled post-treatment effect size of Cohen's *d* = 0.65 [95 % CI 0.56–0.75] with low to negligible levels of heterogeneity (*I*^2^ = 0 % [95 % CI 0 %–9 %]).

### 3.7 Post-hoc analyses of outcomes assessing exhaustion and insomnia

Results from the post-hoc analyses regarding symptoms of exhaustion and insomnia are presented in Figs. S4 and S5 in the Online supplement. Symptoms of exhaustion were assessed in eight studies with nine comparisons and yielded a pooled post-treatment effect size of Cohen's *d* = 0.66 [95 % CI 0.56–0.76] with moderate heterogeneity (*I*^2^ = 60 % [95 % CI 18 %–80 %]). Symptoms of insomnia were assessed in five studies and yielded a pooled post-treatment effect size of Cohen's *d* = 0.49 [95 % CI 0.29–0.70], with a moderate heterogeneity (*I*^2^ = 56 % [95 % CI 19 %–84 %]).

### 3.8 Test of publication bias

Fig. S6 in the Online supplement illustrates the funnel plot based on the main outcome of self-rated stress in the 13 included studies. Egger's test was non-significant (*p* = .34), thus discarding small study bias. Tests of publication bias assuming heterogeneity or homogeneity were not significant *p*'s > .37.

## 4 Discussion

To our knowledge, this is the first meta-analysis to probe the question of whether ICBT can be effective in reducing stress and secondary outcomes of anxiety and depression in targeted samples suffering from elevated perceived stress and stress-related disorders. Even though the number of studies published in the past decade is limited, results from this meta-analysis indicate that ICBT can effectively reduce self-rated perceived stress, anxiety, and depression with moderate to large effect sizes compared to control conditions, with effects on perceived stress that are stable over time. Exploratory post-hoc analyses also indicated that ICBT can be effective to reduce self-reported symptoms of exhaustion and insomnia with moderate effect sizes compared to control conditions.

Previous reviews and meta-analyses of internet-delivered interventions to reduce stress have taken a broad scope and combined a range of interventions, irrespective of theoretical foundation, in the same analyses (Heber et al., 2017; Stratton et al., 2017; Carolan et al., 2017; Ryan et al., 2017; Van Wingerden and Derks-Theunissen, 2018). Further, samples included in previous meta-analyses have ranged from universal or mixed populations that were part of worksite stress-management training programs or employee health-promotion programs (e.g. Umanodan et al., 2014; Billings et al., 2008) to targeted samples with elevated stress, depression and anxiety. This makes it difficult to draw conclusions regarding which type of treatment may be effective for whom, and heterogeneity is often substantial (Heber et al., 2017; Carolan et al., 2017). In the meta-analysis by Heber et al. (2017), that is most similar to the present study regarding aim and scope, six of 23 studies were classified as ICBT and subgroup analyses generated a small effect-size of Cohen's *d* = 0.40 relative to control groups post-intervention. Overall effects on anxiety and depression in that study were small (*d* = 0.32 and 0.34 respectively). In contrast, we found a moderate to large controlled effect size of ICBT for stress-reduction in our meta-analysis of 13 studies (*d* = 0.78), and moderate effects on the secondary outcomes of anxiety and depression (*d* = 0.69 and 0.65 respectively). These effects are more similar to those found in face-to-face CBT for stress-reduction, as indicated by previous meta-analyses in which effects of CBT on self-rated stress ranged from Cohen's *d* = 0.68 to *d* = 1.16 (van der Klink et al., 2001; Richardson and Rothstein, 2008). Even though the present meta-analysis included samples that ranged from elevated perceived stress to clinically diagnosed stress-related disorders, the larger overall effects as compared to earlier meta-analyses might be explained by the fact that only targeted, as opposed to universal, samples were included. Universally recruited samples likely report less pronounced symptoms upon commencing treatment, with limited room for improvement. This being said, previous evidence regarding the effect of stress-management interventions in targeted vs. universal samples has been mixed (Stratton et al., 2017; Amanvermez et al., 2022).

One aspect that makes it difficult to directly compare effects with previous meta-analyses is that interventions are classified in different ways. For example, several of the studies that were included as CBT in the current meta-analysis (e.g. Ebert et al., 2016a; Ebert et al., 2016b; Heber et al., 2016) were classified as “Third-wave cognitive therapies” (TWC) in the meta-analysis of Heber et al (Heber et al., 2017) and as “stress-management” in a meta-analysis by Stratton et al (Stratton et al., 2017). Clearly, the issue of how to classify interventions is important for the compilation and comparison of evidence for a specific type of intervention. Seeing the relatively limited number of studies that have investigated the effect of any type of cognitive or behavioral interventions for targeted samples reporting elevated stress or stress-related disorders, we find it a strength of the present study to include these in the same meta-analysis and systematically report intervention characteristics of these studies. Our findings indicate that treatment components included in the respective interventions are largely similar, which suggests that the classification of cognitive- and behavioral interventions into other categories at this point only risks slowing the compilation of evidence for CBT. When more studies have been conducted, however, subgroup analyses (and eventually meta-analyses) of different subcategories of CBT (e.g., Acceptance and Commitment Therapy, Dialectical Behavior Therapy, Rational Emotive Behavior Therapy) can be studied, as well as subgroup analyses of delivery format and type of support.

### 4.1 Strengths and limitations

It is a strength of this study to specifically investigate the effect of ICBT in targeted samples with elevated perceived stress or stress-related disorders for whom no established treatment guidelines exist to date. The study provides a detailed description of the content and delivery format of internet-delivered interventions based on CBT, which can guide clinicians and researchers in the development and implementation of safe and highly accessible psychological treatment to reduce the clinical impact of stress. This being said, the study has some limitations: Cut-offs on stress scales that were often used for inclusion varied across studies and do not necessarily pinpoint a clinical population. Further, less than half (*n* = 6) of studies recruited participants with stress-related disorders, and only two studies recruited based on clinical referral. Rate of sick leave was very low across studies, indicating that functional disability in study participants was likely limited. Hence, even though our initial ambition was to investigate the efficacy of ICBT in more clinical populations relative to previous meta-analyses, generalizations to clinical samples in healthcare settings should be made with caution. On the same note, only five studies used clinical assessment for inclusion that might allow for identification of other psychiatric disorders commonly associated with elevated stress, such as anxiety disorders or depression (Uliaszek et al., 2012; Liu and Alloy, 2010). Indeed, the population suffering from elevated stress and stress-related disorders remains difficult to operationalize in a consistent way. This is a well-known challenge within the research field of chronic stress and stress-related disorders (Eurofound, 2018; Neckel et al., 2017). Another limitation was that the small number of included studies obstructed reliable subgroup analyses that might have shed light on potential moderating factors of treatment effect. Also, we were unable to conduct separate analyses for studies using waitlist control conditions versus active control conditions given that the latter were only used in three studies. The dominant use of waitlist control conditions might generate measurement bias and overstate treatment effects (Cunningham et al., 2013). When more studies using active control conditions have been conducted, the comparability of effects between trials with different control groups needs to be addressed. Lastly, five of 13 studies were conducted by the same research group, which might introduce some bias, and few studies have been replicated.

### 4.2 Avenues for future research

The promising results of this meta-analysis indicate a need to systematically continue investigation into the effect of CBT to reduce elevated perceived stress and symptoms of stress-related disorders. More studies should investigate treatment effect for clinically referred samples diagnosed with stress-related disorders, given that these are often associated with significant individual suffering, functional impairment, and increased healthcare consumption. Further, more well-designed and well-powered studies are needed to enable subgroup analyses and investigation into potential mediators and moderators of treatment effects. Preregistration of all studies, adherence to the intention-to-treat principle, and adequate methods to account for missing data are important to reduce bias in coming trials. Even though ICBT is an efficient way to reach out to afflicted individuals and the treatment format enables high treatment accessibility, more future studies should compare ICBT for stress-reduction to other active treatments and to face-to-face CBT. Preferably, effectiveness trials should precede full-scale implementation into clinical settings to reduce selection bias.

Even though all studies included in this review and meta-analysis were categorized as cognitive- and/or behavioral interventions, they often consist of a package of different components ranging from, for example, relaxation strategies to problem-solving and coping skills. Compilation of evidence might benefit from more well-described theoretical underpinnings of specific treatment components regarding, for example, in what way they are believed to affect triggers or maintaining factors of stress. Motivating the putative contribution of different treatment components might lead to the development of more theoretically stringent treatment protocols. This is important given that previous meta-analyses indicated that interventions with fewer treatment components may be more efficacious (Richardson and Rothstein, 2008). Further, investigation into whether the effect of ICBT is generalizable also to individuals with specific stressors or psychosocial circumstances is of importance. The present study focused on elevated perceived stress and stress-related disorders and did not include studies that investigated interventions aimed at alleviating stress in specific groups identified based on a certain stressor (such as caregivers of individuals with dementia or parents of children with neuropsychiatric disorders). Whether individually tailored ICBT can meet the needs of specific populations (including those with comorbid psychiatric and somatic conditions), is an important avenue for future research.

Lastly, the Perceived Stress Scale is the most commonly used self-assessed outcome in studies of psychological stress (Lee, 2012), and outcomes of anxiety and depression are usually added. However, capturing the complexity of elevated stress and stress-related disorders may need new outcome measures or at least an agreed-upon core outcome set (COS) that can be used systematically across trials investigating stress-related disorders to facilitate comparisons and compilation of results (Williamson et al., 2012). This is important also because of recent findings that the relative effect of interventions depends on the type of outcome used (Estevez Cores et al., 2021). As a complement to assessing treatment effect on symptom reduction, more objective measures of disability may be used, such as absenteeism and sick leave based on insurance register data. Importantly, when assessing the potential implementation of a new intervention, factors such as treatment adherence, treatment satisfaction, and adverse events related to treatment should systematically be reported.

### 4.3 Conclusions

Seeing that elevated stress and stress-related disorders are recognized as a significant burden to individuals and society at large, the lack of internationally agreed-upon treatment guidelines is striking. The results of this meta-analysis hold important implications for the development of evidence-based clinical guidelines in the face of rapid digital development that can contribute to increased accessibility to care.

## Funding

This research did not receive any specific grant from funding agencies in the public, commercial, or non-profit sectors.

## Declaration of competing interest

The authors declare that they have no known competing financial interests or personal relationships that could have appeared to influence the work reported in this paper.

## Appendix A Supplementary data

Online supplement Image 1
