# Supplementary material for: Internet-delivered cognitive behavioral interventions to reduce elevated stress: A systematic review and meta-analysis
Source: Internet Interv. 2022 Jun 22;29:100553. doi: 10.1016/j.invent.2022.100553 (PMC9240371; doi:10.1016/j.invent.2022.100553)
Supplement: Supplementary file 1 — Online supplement [file mmc1.docx]

**Online supplement**

Table S1: Full search strategy
Table S2: Participant characteristics
Table S3: Interventions characteristics of included studies

Figure S1: Risk of bias summary
Figure S2: Forest plot (secondary outcome of self-rated anxiety)
Figure S3: Forest plot (secondary outcome of self-rated depression)
Figure S4: Forest plot (post-hoc analysis, secondary outcome of self-rated exhaustion)
Figure S5: Forest plot (post-hoc analysis, secondary outcome of self-rated insomnia)
Figure S6: Funnel plot (publication bias)

**Table S1**: Full search strategy as used in the three databases

| **Search Strategy** | **PubMed** | **Web of science** | **PsycInfo** |
| --- | --- | --- | --- |
| 1 | Stress, Psychological [MeSH Terms] | TS= “Stress” | (Map terms) exp stress/ Stress.ti,ab. |
| 2 | Adjustment  Disorders[MeSH Terms] | TS= “Adjustment order” | Adjustment Disorders.ti,ab. |
| 3 | Burnout, Psychological[MeSH Terms] | TS= “Burnout” | Burnout.ti,ab. |
| 4 | Burnout, Professional[MeSH Terms] | TS= “Asthenia” | R Asthenia.ti,ab. |
| 5 | Asthenia[MeSH Terms] | TS= “Neurasthenia” | Neurasthenia.ti,ab |
| 6 | Neurasthenia[MeSH Terms] | TS= “Occupational Stress | Occupational Stress.ti,ab. |
| 7 | Occupational Stress[MeSH  Terms] | TS= “Distress” | Distress.ti,ab. |
| 8 | Stress[Title/Abstract] | TS= “Exhaustion disorder” | Exhaustion disorder.ti,ab. |
| 9 | Distress[Title/Abstract] | TS= “Stress disorder” | Stress disorder.ti,ab. |
| 10 | Adjustment disorder[Title/Abstract] | TS= “Chronic stress” | Chronic stress.ti,ab. |
| 11 | Exhaustion  disorder[Title/Abstract] | TS= “Clinical stress” | Clinical stress.ti,ab. |
| 12 | Stress disorder[Title/Abstract] | TS= “Mental fatigue” | Mental fatigue.ti,ab. |
| 13 | Chronic  stress[Title/Abstract] | TS= “Burn out” | Burn out.ti,ab. |
| 14 | Clinical stress[Title/Abstract] | TS= “Myasthenia” | Myasthenia.ti,ab. |
| 15 | Mental  fatigue[Title/Abstract] | TS= “Work related illnesses” | Work related illnesses.ti,ab. |
| 16 | Burn out[Title/Abstract] | TS= “work related stress“ | work related stress.ti,ab. |
| 17 | Work related  stress[Title/Abstract] | TS= “Psychological stress” | Psychological stress.ti,ab. |
| 18 | Burnout[Title/Abstract | 1 or 2 or 3 or 4 or 5 or 6 or 7 or 8 or 9 or 10 or 11 or 12 or 13 or 14 or 15 or 16 or 17 | 1 or 2 or 3 or 4 or 5 or 6 or 7 or 8 or 9 or 10 or 11 or 12 or 13 or 14 or 15 or 16 or 17 |
| 19 | Asthenia[Title/Abstract] | TS= “Randomized Controlled Trial” | (Map term) randomized  controlled trial Randomized Controlled Trial.ti,ab. |
| 20 | Myasthenia[Title/Abstract] | TS= “Random*” | Random*.ti,ab. |
| 21 | Neurasthenia[Title/Abstract] | TS= “RCT” | RCT.ti,ab. |
| 22 | Occupational stress[Title/Abstract] | TS= “Control” | R Control.ti,ab. |
| 23 | Work related illnesses[Title/Abstract] | TS= “Compare” | 19 or 20 or 21 or 22 |
| 24 | Psychological  stress[Title/Abstract] | 19 or 20 or 21 or 22 or 23 | Compare.ti,ab. |
| 25 | 1 or 2 or 3 or 4 or 5 or 6 or 7 or 8 or 9 or 10 or 11 or 12 or 13 or 14 or 15 or 16 or 17 or 18 or 19 or 20 or 21 or 22 or 23 or 24 | TS= “internet based Intervention” | Internet-Based  Intervention.ti,ab. |
| 26 | Randomized Controlled Trial[MeSH Terms] | TS= “internet” | internet.ti,ab. |
| 27 | Random*[Title/Abstract] | TS= “Web” | Web.ti,ab. |
| 28 | RCT[Title/Abstract] | TS= “Web-based” | web based.ti,ab |
| 29 | Control[Title/Abstract] | TS= “Online” | Online.ti,ab. |
| 30 | Compare[Title/Abstract] | TS= “Computer” | Computer.ti,ab. |
| 31 | 26 or 27 or 28 or 29 or 30 | TS= “Computerised” | Computerised.ti,ab. |
| 32 | Internet Based Intervention[MeSH Terms] | TS= “Computer guided” | Computer guided.ti,ab. |
| 33 | internet[MeSH Terms] | TS= “Smartphone” | Smartphone.ti,ab. |
| 34 | Internet[Title/Abstract] | TS= “App” | App.ti,ab |
| 35 | Web[Title/Abstract] | TS= “Apps” | Apps.ti,ab. |
| 36 | Web based[Title/Abstract] | TS= “e-health” | e-health.ti,ab. |
| 37 | Online[Title/Abstract] | TS= “ehealth” | ehealth.ti,ab. |
| 38 | Computer[Title/Abstract] | 25 or 26 or 27 or 28 or 29 or 30 or 31 or 32 or 33 or 34 or 35 or 36 or 37 | 24 or 25 or 26 or 27 or 28 or 29 or 30 or 31 or 32 or 33 or 34 or 35 or 36 or 37 |
| 39 | Computerised[Title/Abstract] | TS= “Cognitive Behavioral Therapy” | (Map term) Cognitive Behavioral Therapy, Cognitive Behavioral  Therapy.ti,ab. |
| 40 | Computer guided[Title/Abstract] | TS= “Behavior Therapy” | Behavior Therapy.ti,ab. |
| 41 | Smartphone[Title/Abstract] | TS= “Psychotherapy” | Psychotherapy.ti,ab. |
| 42 | App[Title/Abstract] | TS= “Cognitive” | Cognitive.ti,ab. |
| 43 | Apps[Title/Abstract] | TS= “Behavio*” | Behavio*.ti,ab. |
| 44 | ehealth[Title/Abstract] | TS= “CBT” | CBT.ti,ab. |
| 45 | Internet-based  intervention[Title/Abstract] | TS= “ICBT” | ICBT.ti,ab. |
| 46 | 32 or 33 or 34 or 35 or 36 or 37 or 38 or 39 or 40 or 41 or 42 or 43 or 44 or 45 | TS= “Stress management” | Stress management.ti,ab. |
| 47 | Cognitive Behavioral  Therapy[MeSH Terms] | 39 or 40 or 41 or 42 or 43 or 44 or 45 or 46 | 39 or 40 or 41 or 42 or 43 or 44 or 45 or 46 |
| 48 | Behavior Therapy[MeSH Terms] | 18 AND 24 AND 38 AND 47 | 18 AND 23 AND 38 AND 47 |
| 49 | (Psychotherapy[MeSH Terms] | - | - |
| 50 | (Cognitive[Title/Abstract] | - | - |
| 51 | Behavio*[Title/Abstract] | - | - |
| 52 | CBT[Title/Abstract] | - | - |
| 53 | ICBT[Title/Abstract] | - | - |
| 54 | Stress management[Title/Abstract] | - | - |
| 55 | 47 or 48 or 49 or 50 or 51 or 52 or 53 or 54 | - | - |
| 56 | 25 AND 31 AND 46 AND 55 | - | - |
| **Number of hits** | **1694** | **2960** | **1003** |

*Note:* Searches conducted June/July 2020 & May 2021
Overall limits (filter): published between 2010-2020 (2010-2021 in updated search)
Field labels: *=truncation of word for alternate endings; ti,ab. = Title, abstract; TS =Topic
The search was conducted with assistance from research librarian Sabina Gillsund at the Karolinska University Library.

**Table S2**. Participant characteristics of studies included in the meta-analysis.

| Study | Recruitment | Condition | Main inclusion criteria | Main exclusion criteria | Mean age | Women (%) | Education, %** | | | Sick leave (%) |
| --- | --- | --- | --- | --- | --- | --- | --- | --- | --- | --- |
|  |  |  |  |  |  |  | Low | Middle | High |  |
| Ebert et al (2016)^1^ | Occupational health care and general population | Elevated stress | PSS-10≥22; Employed | Psychosis; suicidal ideation | 43 | 86 | 2 | 26 | 72 | 1 |
| Ebert et al (2016)^2^ | Occupational health care and general population | Elevated stress | PSS-10≥22; Employed | Psychosis; suicidal ideation | 42 | 72 | 4 | 29 | 67 | 2 |
| Eimontas et al (2018)^3^ | Facebook page of university | Adjustment disorder | Exposure to a significant life-stressor during the last two years; high level of Adjustment disorder symptoms (according to ADNM-8) | No info | 33 | 82 | No info | 35 | 65 | No info |
| Harrer et al (2018)^4^ | Via university | Elevated stress | PSS-4≥8; university student | Dissociative symptoms or psychosis;  Suicidal ideation | 24 | 75 | 0 | 0 | 100 | No info |
| Heber et al (2016)^5^ | Advertisement general working population | Elevated stress | PSS-10≥22 | Psychosis; suicidal ideation | 43 | 73 | 2 | 21 | 77 | 1 |
| Jonas et al (2016)^6^ | Advertisement | Burnout | ≥3.5 on MBI-emotional exhaustion or MBI-cynicism scales; Employed | Current psychological treatment; sick leave >12 months; high alcohol consumption | 47 | 49 | 0 | 33 | 67 | No info |
| Leterme et al (2020)^7^ | Clinical referral | Adjustment disorder w/anxiety* | Ambulatory patient; Adjustment disorder w/anxiety; no ongoing treatment; stable medication | Pregnancy; other Psychiatric disorder | 38 | 65 | No info | No info | No info | No info |
| Lindsäter et al (2018)^8^ | National, via advertisement | Adjustment disorder or exhaustion disorder* | Diagnosis of adjustment disorder or exhaustion disorder | Psychosis; suicidal ideation; ongoing psychological treatment; changed medication in past month | 46 | 85 | 9 | 18 | 73 | 14 |
| Mehring et al (2016)^9^ | Referral by general practitioners | Elevated stress* | Desire for stress-reduction | Current or past psychiatric disorder | 42 | 59 | No info | No info | No info | No info |
| Nixon et al (2021)^10^ | Advertisement | Elevated stress | Employed; PSS-10 ≥22 | Psychosis; suicidal ideation | 42 | 77 | 3 | 26 | 71 | 2 |
| Persson-Asplund et et al (2018)^11^ | Advertisement | Adjustment disorder; Reaction to severe stress* | Stress-disorder included in F43 section in ICD-10; employed as middle manager; | Full-time sick leave; score of <25 on the PSS-14 and <2.75 on the SMBQ. | 47 | 67 | No info | No info | 91 | 1 |
| Rachyla et al (2020)^12^ | Advertisement | Adjustment disorder* | Adjustment disorder | Suicide risk or self-harm; severe mental disorder; changed medication; other psychological treatment for adjustment disorder | 33 | 75 | 8 | 17 | 75 | 3 |
| Rose et al (2013)^13^ | Advertisement to graduate students | Elevated stress | PSS-10>16 | Psychiatric disorder | 27 | 50 | 0 | 0 | 100 | No info |

*Note:* *Clinical assessment was conducted. **Highest level of education was operationalized differently across studies but generally corresponds to “Low”: primary school; “Middle”: High school; “High”: College/university studies.

PSS, Perceived Stress Scale; ADNM, the Brief Adjustment Disorder New Module; ICD-10, International Classification of Diseases, 10^th^ edition; MBI, Maslach Burnout Inventory; HADS, Hospital Anxiety and Depression scale SMBQ; Shirom-Melamed Burnout Questionnaire

**Table S3**. Selected intervention characteristics of included studies.

| **Study** | **Main content** | **Lable** | **Guidance^a^** | **Delivery** | **Length (weeks)** |
| --- | --- | --- | --- | --- | --- |
|  |  |  |  |  |  |
| Ebert et al (2016)^42^ | Psychoeducation, problem-solving, emotion regulation, plan for the future, booster session. Optional content integrated into pre-selected modules: Time management, rumination and worrying, psychological detachment from work, sleep hygiene, rhythm and regularity of sleeping habits, nutrition and exercise, organization of breaks during work, social support. | GET.ON Stress | AFG | Web/ mobile | 7 |
| Ebert et al (2016)^41^ | Same content as Ebert et al 2016^42^ | GET.ON Stress | CAG | Web/mobile | 7 |
| Eimontas et al (2018)^50^ | Physical relaxation, Time management, Mindfulness, Strengthening relationships | The Brief Adjustment Disorder Intervention (BADI) | SG | Web | 4 |
| Harrer et al (2018)^44^ | Same content as Ebert et al 2016^42^ with the addition of third-wave CBT: Mindfulness, Acceptance and Tolerance, Self-Compassion (including cognitive restructuring) | StudiCare Stress (based on GET.ON Stress) | AFG | Web/mobile | 7 |
| Heber et al (2016)^43^ | Same content as Ebert et al 2016^42^ | GET.ON Stress | AFG | Web/mobile | 4-7 |
| Jonas et al (2016)^45^ | Stress and relaxation diary, Minimization of existing strains, Develop coping behavior, Challenging perfectionism and unrealistic standards, identifying goals and values, Practice relaxation and pleasure, Seek assistance and social support | Beratung Hilft (counseling helps)^b^ | WG | Web | 4 |
| Leterme et al (2020)^40^ | Psychoeducation, Body relaxation, Cognitive therapy, Mindfulness, Exposure and positive attitudes | Seren@ctif: | AFG | Computer (with physical start-up and finish for each module) | 5 |
| Lindsäter et al (2018)^49^ | Psychoeducation, recovery training, sleep hygiene, life values, behavioral activation, exposure, cognitive reappraisal, communication skills and assertiveness-training, relapse-prevention | Gustavsbergs Primary care CBT for stress-related disorders | WG | Web | 12 |
| Mehring et al (2016)^47^ | Identify personal stress patterns, calmness/relaxation strategies, release tension and recharge, grief, psychoeducation, balance, “stress-free in the workplace”, coping strategies | WeCARE^c^ GmbH | CAG | Web | 12 |
| Nixon et al (2021)^46^ | (same content as Ebert et al 2016a) | GET.ON Stress | AFG/SG | Web | 4-7 |
| Persson-Asplund et al (2018)^48^ | Psychoeducation, problem-solving, behavioral activation, life balance (e.g., recovery techniques, applied relaxation, work-home interface), exposure (e.g., assertiveness training, perfectionism, worry), positive management (positive and corrective feedback). Optional content: sleep management | iStress | WG | Web | 8 |
| Rachyla et al (2020)^52^ | Motivation for change, psychoeducation, behavior activation, a slow breathing technique, exposure, problem-solving, mindfulness, development of personal strengths and optimism towards the future, relapse prevention. | TAO (Trastornos Adaptativos Online). | WG | Web | 7-10 |
| Rose et al (2013)^51^ | Psychoeducation, breathing and relaxation exercises, emotion/physiological regulation skills, cognitive flexibility, actions to manage stress in their lives | SMART-OP | RO | Web and Computer | 6 |

^a^AFG: Adherence-focused guidance; WG: Weekly guidance; CAG: Computer-automated guidance; SG: self-guided; RO: self-guided with reminders via mail or telephone

^b^The program is initiated and completed with a 50-minute individual chat session with the therapist

^c^Participants could also communicate with one another through a forum or ask a medical team member in case they had any questions


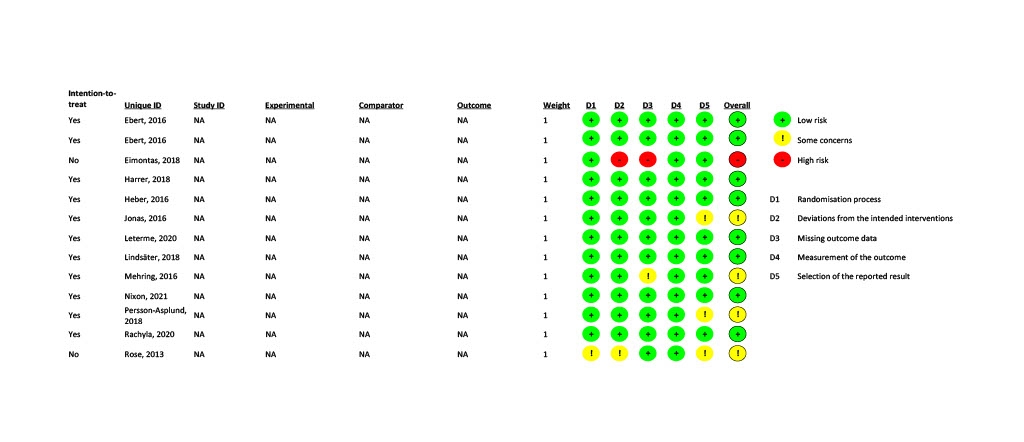
**Fig S1.** Risk of bias summary of studies included in the meta-analysis, based on five dimensions of the revised Cochrane risk of bias tool for randomized trials (Rob2). Risk of bias was assessed as follows: (D1) Non-random allocation to the respective groups; (D2) Possibility of changes to the trial protocol that might have affected the outcome of the results; (D3) If missining data might affect the results of the study (e.g., more than 15% attrition); (D4) If the method to measure the outcome is appropriate; (D5) If data was analyzed in accordance to a pre-specified plan.


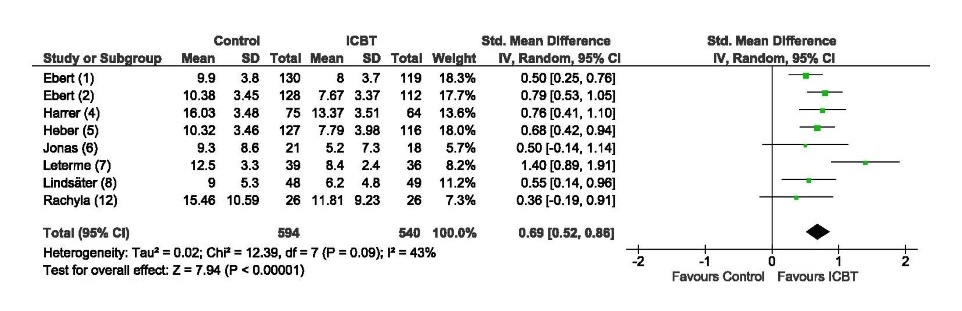


**Fig S2**. Forest plot of post-intervention effect sizes of included Internet-based cognitive behavioral interventions (ICBT) compared with control conditions, on the secondary outcome of self-rated anxiety. References are given in parentheses.


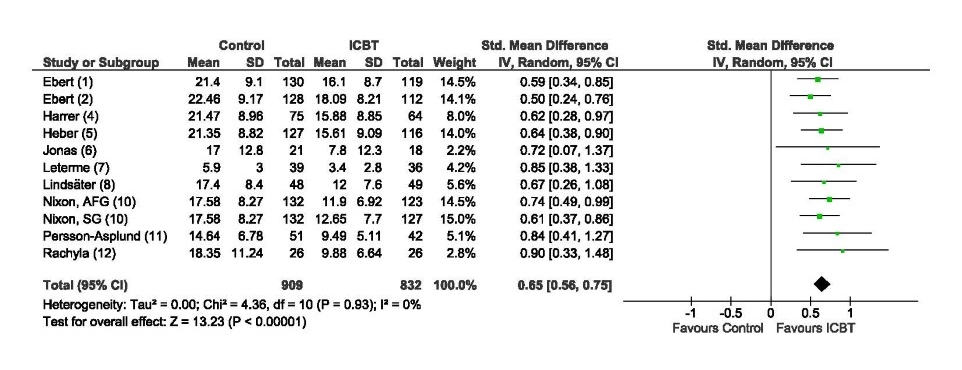


**Fig S3**. Forest plot of post-intervention effect sizes of included Internet-based cognitive behavioral interventions (ICBT) compared with control conditions, on the secondary outcome of self-rated depression. AFG, Adherence focused guidance. SG, Self-guided. References are given in parentheses.


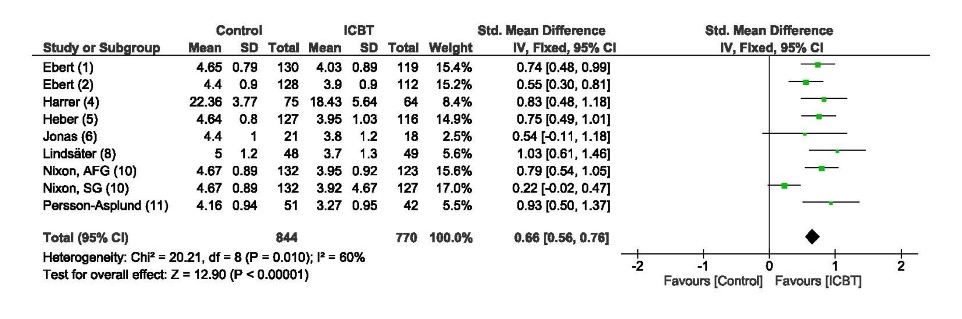


**Fig S4**. Forest plot of post-intervention effect sizes of included Internet-based cognitive behavioral interventions (ICBT) compared with control conditions, in the post-hoc analysis of the secondary outcome of self-rated exhaustion. AFG, Adherence focused guidance; SG, Self-guided. References are given in parentheses.


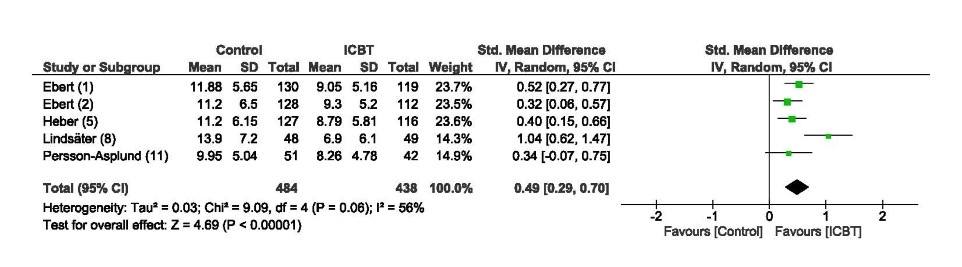


**Fig S5**. Forest plot of post-intervention effect sizes of included Internet-based cognitive behavioral interventions (ICBT) compared with control conditions, in the post-hoc analysis of the secondary outcome of self-rated insomnia. References are given in parentheses.


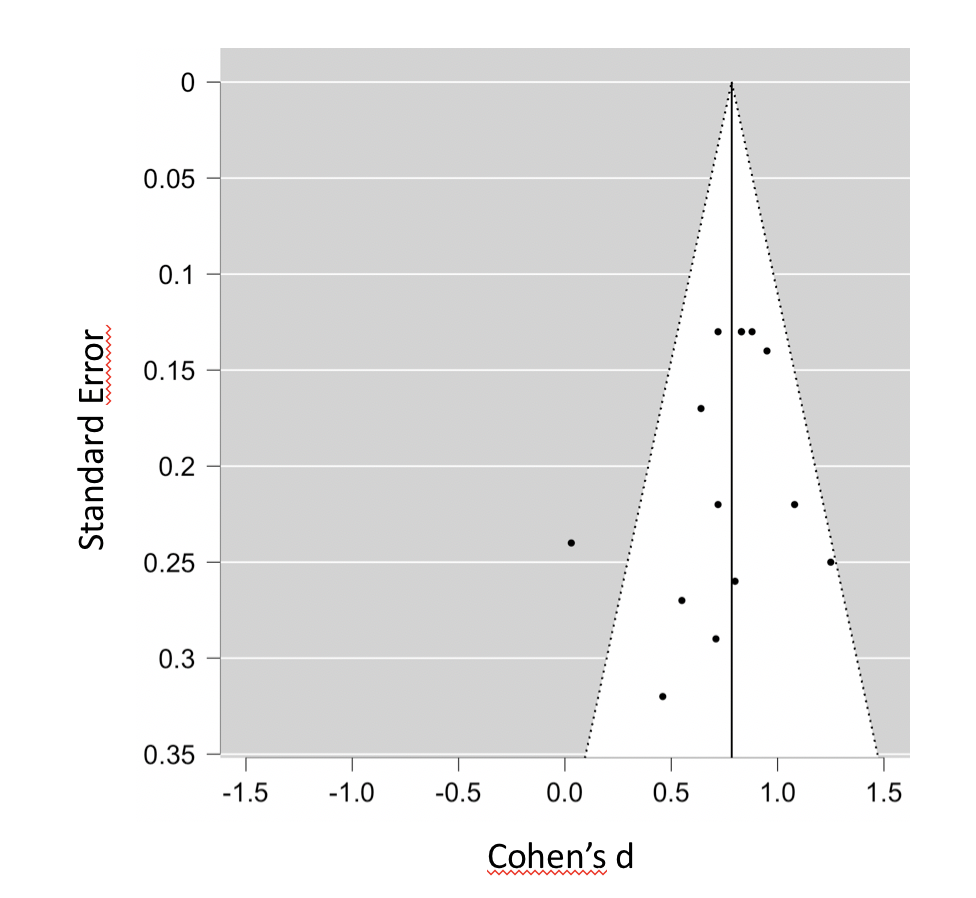


**Fig S6.** Funnel plot of studies investigating the effect of internet-delivered cognitive behavioral thearapy for stress. The risk of publication bias is typically assumed to be lower if spread around the mean effect size (black line) decreases along with the standard error.

**REFERENCES**

1. Ebert DD, Lehr D, Heber E, Riper H, Cuijpers P, Berking M. Internet- and mobile-based stress management for employees with adherence-focused guidance: efficacy and mechanism of change. *Scand J Work Environ Health* 2016; **42**: 382-94.

2. Ebert DD, Heber E, Berking M, Riper H, Cuijpers P, Funk B, et al. Self-guided internet-based and mobile-based stress management for employees: results of a randomised controlled trial. *Occup Environ Med*. 2016; **73**: 315-23.

3. Eimontas J, Rimsaite Z, Gegieckaite G, Zelviene P, Kazlauskas E. Internet-Based Self-Help Intervention for ICD-11 Adjustment Disorder: Preliminary Findings. *Psychiatr Q*. 2018; **89**: 451-60.

4. Harrer M, Adam SH. Effectiveness of an Internet- and App-Based Intervention for College Students With Elevated Stress: Randomized Controlled Trial. 2018; **20**: e136.

5. Heber E, Lehr D, Ebert DD, Berking M, Riper H. Web-Based and Mobile Stress Management Intervention for Employees: A Randomized Controlled Trial. *Journal of Medical Internet Research*. 2016; **18**: e21.

6. Jonas B, Leuschner F, Tossmann P. Efficacy of an Internet-based Intervention for Burnout: A randomized controlled trial in the German Working Population. *Anxiety Stress Coping*. 2016: 1-27.

7. Leterme A, Behal H, Demarty A, Barasino O, Rougegrez L, Labreuche J, et al. A blended cognitive behavioral intervention for patients with adjustment disorder with anxiety: a randomized controlled trial. *Internet interventions*. 2020; **21**: 100329.

8. Lindsäter E, Axelsson E, Salomonsson S, Santoft F, Ejeby K, Ljotsson B, et al. Internet-Based Cognitive Behavioral Therapy for Chronic Stress: A Randomized Controlled Trial. *Psychother Psychosom*. 2018; **87**: 296-305.

9. Mehring M, Haag M, Linde K, Wagenpfeil S, Schneider A. Effects of a Web-Based Intervention for Stress Reduction in Primary Care: A Cluster Randomized Controlled Trial. *J Med Internet Res*. 2016; **18**: e27.

10. Nixon P, Boß L, Heber E, Ebert DD, Lehr D. A three-armed randomised controlled trial investigating the comparative impact of guidance on the efficacy of a web-based stress management intervention and health impairing and promoting mechanisms of prevention. *BMC Public Health*. 2021; **21**: 1-18.

11. Persson Asplund R, Dagöö J, Fjellstrom I, Niemi L, Hansson K, Zeraati F, et al. Internet-based stress management for distressed managers: results from a randomised controlled trial. *Occup Environ Med*. 2018; **75**: 105-13.

12. Rachyla I, Perez-Ara M, Moles M, Campos D, Mira A, Botella C, et al. An internet-based intervention for adjustment disorder (TAO): study protocol for a randomized controlled trial. *BMC Psychiatry*. 2018; **18**: 161.

13. Rose RD, Buckey JC, Jr., Zbozinek TD, Motivala SJ, Glenn DE, Cartreine JA, et al. A randomized controlled trial of a self-guided, multimedia, stress management and resilience training program. *Behav Res Ther*. 2013; **51**: 106-12.
